# Genome-wide analysis on the maize genome reveals weak selection on synonymous mutations

**DOI:** 10.1186/s12864-020-6745-3

**Published:** 2020-04-29

**Authors:** Duan Chu, Lai Wei

**Affiliations:** College of Life Sciences, Beijing Normal University, No. 19 Xinjiekouwai Street, Haidian District, Beijing, China

**Keywords:** Synonymous mutations, tRNA adaptation index (tAI), Maize (*Zea mays*), Derived allele frequency (DAF), Minor allele frequency (MAF), Natural selection

## Abstract

**Background:**

Synonymous mutations are able to change the tAI (tRNA adaptation index) of a codon and consequently affect the local translation rate. Intuitively, one may hypothesize that those synonymous mutations which increase the tAI values are favored by natural selection.

**Results:**

We use the maize (*Zea mays*) genome to test our assumption. The first supporting evidence is that the tAI-increasing synonymous mutations have higher fixed-to-polymorphic ratios than the tAI-decreasing ones. Next, the DAF (derived allele frequency) or MAF (minor allele frequency) of the former is significantly higher than the latter. Moreover, similar results are obtained when we investigate CAI (codon adaptation index) instead of tAI.

**Conclusion:**

The synonymous mutations in the maize genome are not strictly neutral. The tAI-increasing mutations are positively selected while those tAI-decreasing ones undergo purifying selection. This selection force might be weak but should not be automatically ignored.

## Background

As understood by the broad researchers, synonymous mutations do not change the amino acid (AA) sequences. However, they are still subjected to natural selection [1–3]. For instance, a few synonymous mutations occurring in the proper place could affect mRNA splicing [4, 5]. Another impact of synonymous mutations is the change in tRNA adaptation index (tAI) [6], a terminology which described the tRNA availability of a codon.

For one of the 61 sense codons, its translation rate or decoding rate is largely determined by how many cognate tRNAs are available. Codons with higher tRNA concentrations tend to have higher translation rates. These codons are regarded as optimized codons or optimal codons [7, 8]. It is intuitive to consider that the change in tAI caused by synonymous mutations should undergo selection force. This selection is certainly independent of amino acid sequences because they do not alter the amino acids. Although this notion is verified in a limited number of animal species, the genome-wide situation (selection patterns on synonymous mutations) in plant kingdom remains largely unknown. What we currently know is the following messages and knowledges of the codon bias phenomenon in plant species.

We summarized the recent progresses of codon bias (codon optimality) and its selection patterns in plants. A study in the ancient gymnosperm species *Gingko biloba* found higher frequency for A/T ending codons than G/C ending codons, but meanwhile it found that the highly expressed genes and those genes involved in environmental adaptation tend to use C/G ending codons, suggesting that the Gingko genome is dominated by natural selection [9]. Similarly, in angiosperms, it was found that the G/C ending codons were optimal (e.g. in *Arabidopsis thaliana*) and that there was enrichment of these G/C ending codons in monocots compared to dicots [10]. Another study in four non-grass monocot species also proposed that the G/C ending codons are optimal and that this preference is not likely caused by mutation biases [11]. Nevertheless, a recent study on four cotton species found the pattern that the pyrimidine-enriched codons (especially those ending with T) have higher frequency in the CDSs [12]. Meanwhile, it was also declared that the GC3 (GC content at the 3rd codon position) method was not always suitable for evolutionary comparisons at different scales [13] although CAI is positively correlated with GC3 [11]. From another aspect, the strength of codon bias could be largely different in different species. Previous studies comparing rice and *Arabidopsis* discovered that codon bias is stronger in housekeeping genes [14], and that the preference for increasing CDS GC content is stronger in rice [15].

Maize is a domesticated model plant like rice which has a well-annotated genome [16, 17], and it might be under particular selection mode. Previous works did investigate the codon optimality in many plant species including rice. However, (1) maize is less studied compared to rice although they are both domesticated monocots; (2) few studies associated their conclusions with the selection on translation efficiency because neither GC3 nor CAI (codon adaptation index) could directly measure the translational status (but the translation-related parameter tAI is correlated with CAI or GC3).

In this study, we are going to test our assumption in the maize (*Zea mays*) genome. Following our previous work [18], we extracted the polymorphic mutations in CDS using the public RNA-seq data (Methods). We also used two outgroup species wheat (*Triticum aestivum*) (*Poaceae*) and carrot (*Daucus carota*) (*Apiaceae*) to determine the direction of mutations when necessary.

Our results would reveal the weak selection force acting on synonymous mutations caused by tAI-changing. This selection force seems to be weaker than the strong constrain on missense mutations. However, the mini effect of synonymous changes should not be automatically ignored as in many evolutionary studies. Our current work on synonymous mutations could be appealing to the geneticists, evolutionary biologists as well as phytologists.

## Results

### Mutations in coding regions in maize

**Fig. 1:**
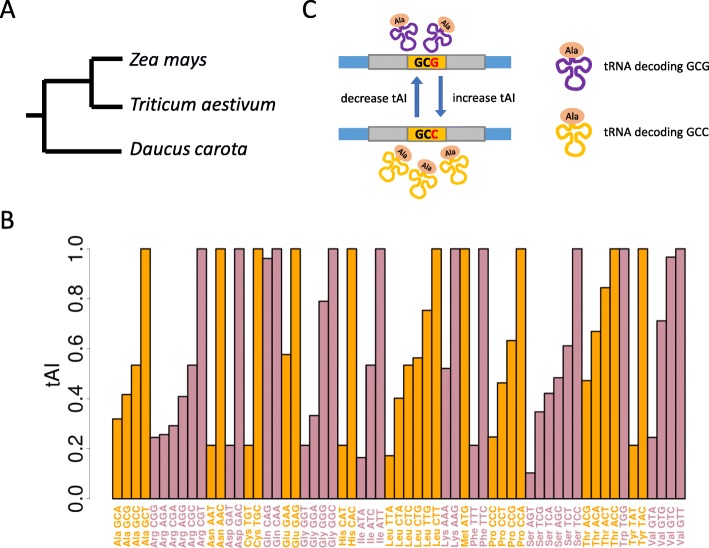
An overview of the methods and materials used in this study. **a** Phylogeny of the plant species used in this study. The branch length is unscaled. **b** tAI of the sense codons in maize. **c** A diagram telling the readers our definition of synonymous mutations that increase or decrease the tAI

According to our recent work [18], the polymorphic sites in CDS were retrieved (Methods). The fixed mutations in CDS are extracted with the CDS alignments between maize and other two outgroup species wheat and carrot (Fig. 1a, Additional file 1: Figure S1). To ensure that the orthologous sites in wheat or carrot are not polymorphic, we downloaded RNA-seq data of roots generated from the corresponding species and mapped the RNA-seq reads to the reference CDS and discarded all the potential polymorphic sites (Methods).

Under these criteria, we obtained 12,041 polymorphic and 843,285 fixed mutation sites in CDS of maize (Additional file 1: Figure S2). Among the 12,041 polymorphic mutations, 4865 are synonymous, 6875 are nonsynonymous and 301 are nonsense mutations. Among the 843,285 fixed mutations, 437,056 are synonymous, 400,943 are nonsynonymous and 5286 are nonsense mutations (Additional file 1: Figure S2).

### Synonymous mutations that increase or decrease the tAI value

We defined the tAI values for each sense codon in maize (Fig. 1b). Within each AA, any single base synonymous mutations that change a low-tAI codon to a high-tAI codon are defined as mutations that increase tAI (e.g. Ala codons GCG to GCC in Fig. 1c), and vice versa (Fig. 1c and Additional file 2: Table S1). Note that the tAI defined by us is also termed “AA tAI” in some literature [19]. This does not affect the classification of mutations because we only compare the relative tAI values within each AA and we do not consider the nonsynonymous mutations (that may also change the tAI values).

Among the 61 sense codons, 87 “codons pairs” could be switched by a single base mutation and cause tAI change (Additional file 2: Table S1). We listed the codon pairs with the direction of increasing tAI in Additional file 2: Table S1 and the opposite direction is decreasing tAI.

### Fixed to polymorphic ratios reveal the weak selection on synonymous mutations

**Fig. 2:**
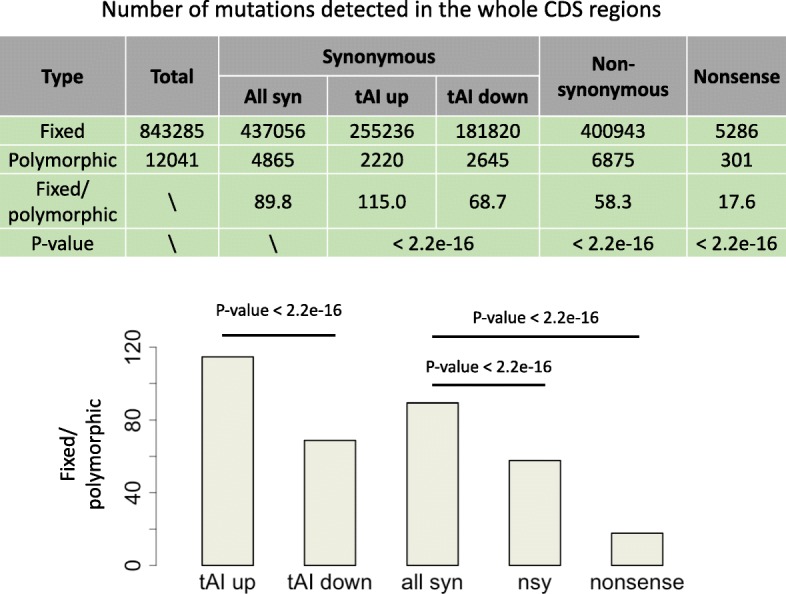
Numbers of fixed and polymorphic mutations of different categories. These categories include synonymous, nonsynonymous and nonsense mutations as well as the synonymous mutations that increase or decrease the tAI. *P* values are calculated from Fisher’s exact tests

It is well established that the nonsynonymous or nonsense mutations are overall deleterious and while the synonymous mutations are regarded as neutral. Usually, the nonsynonymous to synonymous ratios are measurements for the adaptiveness of different sets of nonsynonymous mutations [20, 21]. Accordingly, we observed that the fixed to polymorphic ratios of nonsynonymous or nonsense mutations are significantly lower than that of synonymous mutations (Fig. 2). We wonder whether we could see differences in fixed to polymorphic ratios between the two categories of synonymous mutations.

Among the 4865 polymorphic synonymous mutations, 2220 cause an increase in tAI and 2645 cause a decrease in tAI. Among the 437,056 fixed synonymous mutations, 255,236 increase tAI and 181,820 decrease tAI (Fig. 2). This result means that the synonymous mutations that increase the tAI have significantly higher fixed to polymorphic ratio (115.0) than those mutations that decrease the tAI (68.7) (Fig. 2, Fisher’s exact test), suggesting an overall positive selection for synonymous mutations that increase the tAI or purifying selection on synonymous mutations that decrease the tAI. We should emphasize that the absolute values of the fixed to polymorphic ratios might not be precise because if some polymorphic mutations are omitted due to the limited coverage of RNA-seq data, then this fixed to polymorphic ratio would be overestimated. However, the comparison of their relative values (tAI-up versus tAI-down) are feasible among different categories.

### Derived allele frequency (DAF) spectrum supports the positive selection on synonymous mutations that increase the tAI

**Fig. 3:**
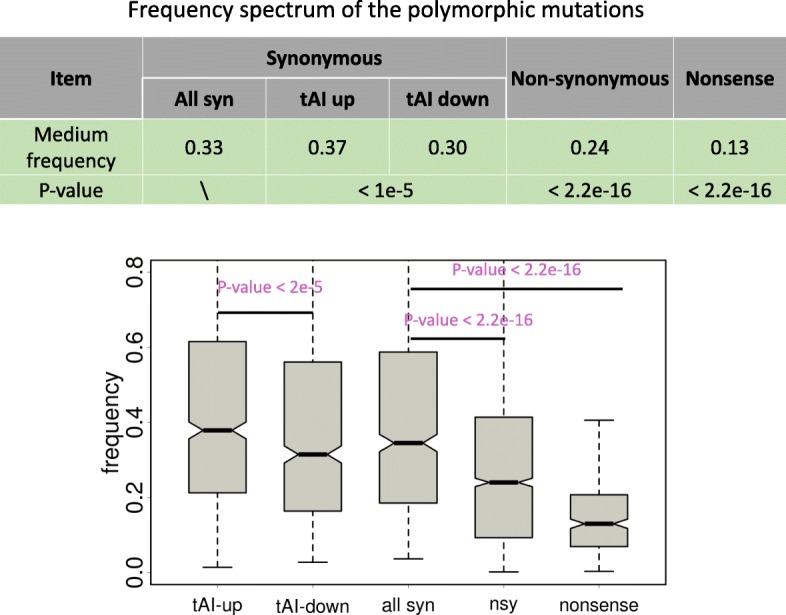
Frequency spectrum of polymorphic mutations of different categories. These categories include synonymous, nonsynonymous and nonsense mutations as well as the synonymous mutations that increase or decrease the tAI. *P* values are calculated from Wilcoxon rank sum tests

We next investigated the derived allele frequencies of the polymorphic mutations. Similar to our analyses in the previous section, we listed and plotted the DAF spectrum of synonymous (tAI up and tAI down, respectively), nonsynonymous and nonsense mutations (Fig. 3). We first verified the known trend that the derived allele frequencies of mutations exhibit nonsense < nonsynonymous < synonymous (Fig. 3, Wilcoxon rank sum tests) due to the overall deleterious effects of nonsynonymous and nonsense mutations. Next, within the synonymous mutations, we found that the tAI-up mutations have significantly higher DAF than the tAI-down mutations (Fig. 3, Wilcoxon rank sum tests), supporting the positive selection for synonymous mutations that increase the tAI and purifying selection on mutations that decrease the tAI.

### The pattern is robust if we look at minor allele frequency (MAF) of all mutations instead of derived allele frequency

In the DAF analysis, in order to ensure the ancestral alleles we found are reliable, we have discarded many sites that show discrepancy between the two outgroup species (Additional file 1: Figure S1). This action might omit some true positive messages and reduce the statistical power. Here we retrieve all the variations in CDS (not considering the ancestral state) and calculate the minor allele frequency (MAF) of each mutation site instead of DAF.

**Fig. 4:**
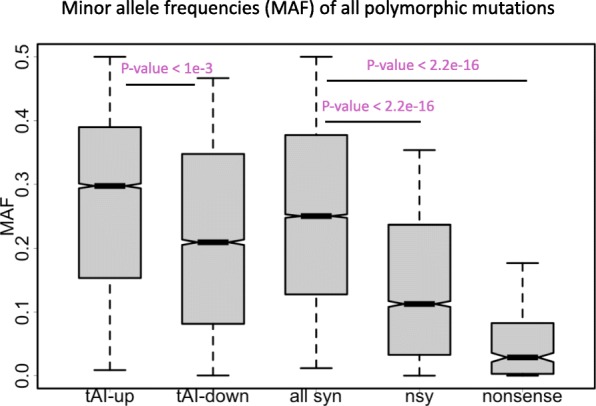
Minor allele frequencies (MAF) of all polymorphic mutations of different categories. These categories include synonymous, nonsynonymous and nonsense mutations as well as the synonymous mutations that increase or decrease the tAI. *P* values are calculated from Wilcoxon rank sum tests

MAF is usually defined as the second most abundant allele on a position. For a bi-allelic site (most cases), MAF is the frequency of the less frequent allele. Thus, MAF should be lower than or equal to 0.5. Here we also first verified the known pattern that the MAF of mutations exhibited nonsense < nonsynonymous < synonymous (Fig. 4, Wilcoxon rank sum tests) due to the purifying selection on nonsynonymous and nonsense mutations. Next, within the synonymous mutations, we found that the tAI-up mutations have significantly higher MAF than the tAI-down mutations (Fig. 4, Wilcoxon rank sum tests), supporting the advantage of synonymous mutations that increase the tAI and the slightly deleterious effect of mutations that decrease the tAI.

### Signals of natural selection are stronger in highly expressed genes

The advantage of RNA-seq is that the data contain information of expression level for each gene. It is well known that highly expressed genes tend to be more conserved and functionally more important than lowly expressed genes. The strength of natural selection is also stronger and more efficient in highly expressed genes. If our observed patterns are truly shaped by natural selection, we should see stronger patterns in highly expressed genes. According to the RNA-seq data in maize, we used RPKM (reads per kilobase per million mapped reads) to measure the expression level of each gene and divided the genes into two groups with high or low RPKM values. Amazingly, in highly expressed genes, we found a greater difference in (1) the fixed to polymorphic ratios (Additional file 1: Figure S3) and (2) DAF and MAF spectrum (Additional file 1: Figure S4) between tAI-up and tAI-down mutation sets. These results again support our hypothesis.

### Similar trends are obtained for CAI instead of tAI

While tAI measures the tRNA accessibility of a codon, CAI directly measures the relative abundance of synonymous codons in the genome. Since both tAI and CAI are frequently used in the studies of codon usage bias, we wonder whether our observed patterns on tAI hold true if we look at CAI.

Precisely speaking, at codon level, the parameters used to measure codon usage are termed RSCU (relative synonymous codon usage) or w_ij_. CAI is the geometric mean of w_ij_ values of a gene (Methods). However, a mutation that increases the w_ij_ value of a codon would definitely increase the CAI of its host gene. Thus, we still use the term CAI to represent the w_ij_ values of codons (Methods).

**Fig. 5:**
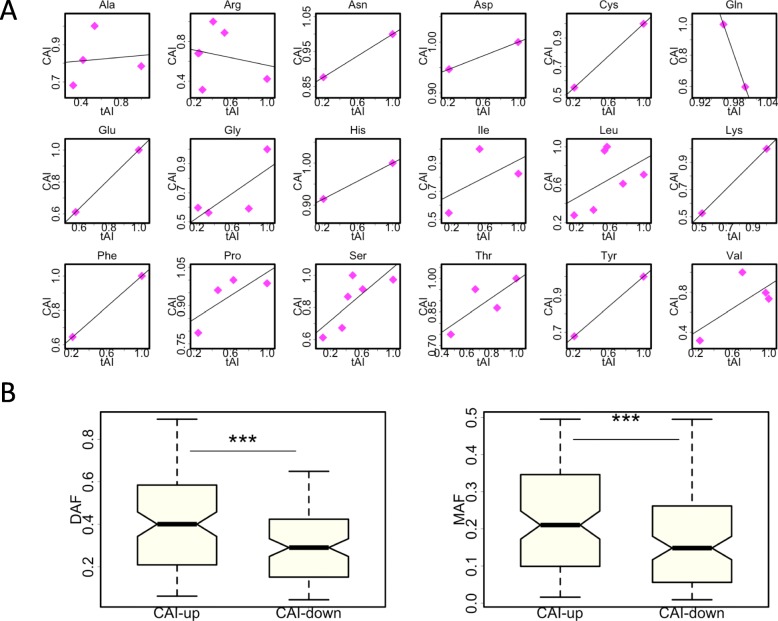
The observed patterns are robust when we investigate CAI (codon adaptation index) instead of tAI. **a** The relationship between tAI and CAI. The 18 amino acids (Met and Trp excluded) are plotted separately. A linear regression line is drawn in each panel. **b** DAF and MAF spectrum of synonymous mutations that increase or decrease the CAI. *P* values are calculated from Wilcoxon rank sum tests

We compared the tAI versus CAI values within synonymous codons (Fig. 5a). Among the 20 amino acids (AAs), Met and Trp do not have synonymous codons and therefore are excluded. For the remaining 18 AAs, we found positive correlation between tAI and CAI for 16 AAs (Fig. 5a). This suggests that the majority of synonymous mutations that increase tAI would also increase CAI, and vice versa. We divided all synonymous mutations into two groups: CAI-up and CAI-down. We found that the CAI-up group had significantly higher DAF as well as MAF than the CAI-down group (Fig. 5b). These results are expected when given the strong correlation between tAI and CAI.

### GC content links tAI with RNA folding

While higher tAI values are suitable for faster decoding during translation, another cis determinant, RNA folding (RNA secondary structure), would intuitively slow down the translating ribosomes. These two factors both impact the translation process at the elongation step, so we asked whether there is interplay between tAI and RNA folding.

As we listed in Additional file 2: Table S1, the synonymous mutations that increase the tAI are usually NNA/T-to-NNG/C mutations. This pattern also exists for CAI because the GC-ending synonymous codons often have higher frequencies in the genome. We observed a strong positive Spearman correlation between gene level GC content and tAI value (Additional file 1: Figure S5a, *p*-value < 2.2e-16). This means that genes with higher GC content have more optimal codons for fast translation.

On the other hand, G/C have stronger base-pairing capability than A/T, so that GC-enriched genes might be more likely to form RNA secondary structures. To test this hypothesis, we employed bioinformatic software to predict and calculate the fraction of RNA structured region (structured%) for a given CDS (Methods). We found a positive Spearman correlation between GC content and “structured%” of a gene (Additional file 1: Figure S5b, *p*-value < 0.001). Moreover, within the secondary structure region, the GC base content is significantly higher than that of the outside (Additional file 1: Figure S5c). Finally, we tested the correlation between tAI and “structured%” of a gene and still found a significantly positive correlation (Additional file 1: Figure S5d, *p*-value <1e-4).

We surmise that the link between tAI and RNA secondary structure might be mediated by the GC content because so far, no evidence shows the direct causality relationship between tAI and RNA folding. However, it is interesting to think about this trade-off associated with GC content. Higher GC contents might lead to higher tAI (faster translation) but meanwhile leading to stronger RNA structure (slower translation).

### The selection on tAI is stronger for genes with higher GC contents

A previous literature [22] has reported that in yeasts the mutations are biased towards AT with nearly three folds while the GC-biased gene conversion (gBGC) [23] would convert the AT alleles to the GC alleles. The expected GC content should be 0.26 but the observed GC3 at degenerate 3rd codon position is 0.38. The observed GC content is a balance between mutation bias and gBGC. Together with our observations above, the genome-wide GC content might have impact on the tAI patterns.

To discuss how gBGC could affect the tAI and our conclusion, we divided the genes into two halves according to GC contents and see whether our observed patterns on tAI still exist. Intriguingly, in genes with higher GC contents, we found a greater difference in DAF or MAF between tAI-up and tAI-down mutation sets (Additional file 1: Figure S6). This pattern resembles the result of highly versus lowly expressed genes. We guess it is possible that the genes with higher GC content are favored (compared to those with lower GC content) and therefore the strength of natural selection is more efficient in these genes.

Furthermore, it was reported that mutations towards GC have a higher probability to be transferred to the next generation and eventually be fixed [24]. The pattern has been observed in the human genome [25]. This bias is possibly due to the GC-biased mismatch repair mechanism and the recombination hotspots. We show our data that the median distance between the nearby tAI-up and tAI-down mutations is less than 90 bp (Additional file 1: Figure S7). However, for recombination hotspots, the “unit” for recombination should be “Kb” or longer. If recombination bias does play a role, it should equally affect the tAI-up and tAI-down mutations, which means that the tAI-up mutations near the hotspots should have higher allele frequencies than the tAI-up mutations far from recombination hotspots. In other words, under recombination bias, the high frequency mutations should be clustered and the low frequency mutations should be clustered, respectively. Take a “tAI-up” mutation for instance, if its nearest mutation is also a “tAI-up” mutation, we name it “up near up”. If its nearest mutation is a “tAI-down” mutation, we name it “up near down”. Similarly, we define “down near up” and “down near down” mutations. We find no difference between the DAF/MAF of “up near up” versus “up near down” groups, or “down near up” versus “down near down” groups (Additional file 1: Figure S8). The high frequency or low frequency mutations are not clustered. This result denies the possibility that the observed difference between “up” and “down” mutations is caused by recombination hotspots.

### Relative contribution of tAI, CAI and GC content to the frequency spectrum

**Fig. 6:**
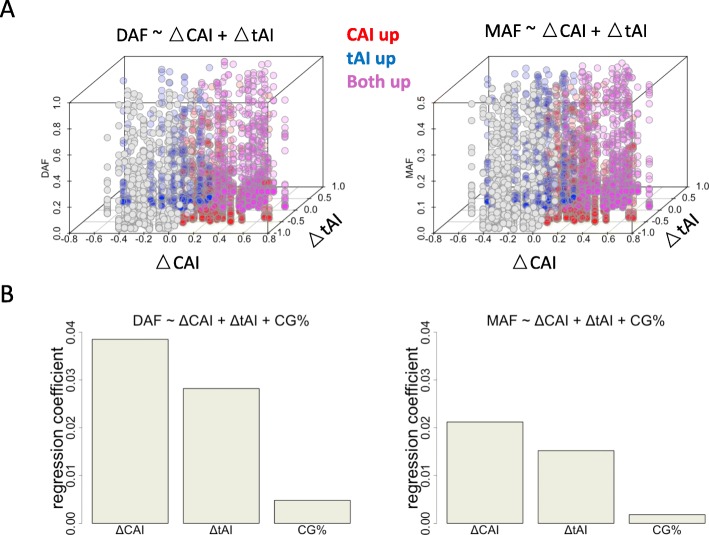
Relative contribution of each feature to the allele frequency spectrum of synonymous mutations. **a** Scatterplot showing the relationship between DAF/MAF and the change of CAI/tAI values. Each dot represents a synonymous mutation. Colors representing different sets of sites are explained next to the plot. **b** Regression coefficients of CAI, tAI and GC content against DAF/MAF

It is certain that multiple factors could affect the observed mutation spectrum, and therefore we perform the multiple regression: Y ~ X1 + X2 + X3. Let’s assume a dataframe with N rows and 4 columns. N is the number of mutation sites. Column 1 (Y) is the DAF or MAF (numeric); Column 2 (X1) is the CAI change (numeric); Column 3 (X2) is the tAI change (numeric); Column 4 (X3) is the GC percentage of the corresponding gene (numeric). Since these variables are all numeric, by performing regression Y ~ X1 + X2 + X3, we would know the relative contribution of each variable. We first plot Y ~ X1 + X2 (Fig. 6a). Both X1 (CAI change) and X2 (tAI change) are positively correlated with Y. Next, we provide the regression coefficients of X1, X2 and X3 against Y (Fig. 6b). The result supports a major role of CAI and tAI, and a minor role of GC percentage. The selection on CAI or tAI is truly playing a role in shaping the allele frequency spectrum.

We have mentioned that tAI is correlated with CAI or GC3. Within each amino acid, we classified high tAI codons and low tAI codons (2 versus 2 for AAs with four codons, and 3 versus 3 for AAs with 6 codons; For Ile, Met and Trp, the numbers were “rounded” towards high tAI codons). Among the 32 high tAI codons, 23 were G/C-ending and 9 were A/T-ending. Among the 29 low tAI codons, 8 were G/C-ending and 21 were A/T-ending. Together with the positive correlation between tAI and CAI, our results agreed with previous literatures which claimed that G/C-ending codons are generally optimal.

## Discussion

Literally, “synonymous” means “the same”. But a bunch of studies already demonstrated that some synonymous mutations affected mRNA splicing [4, 5, 26]. Here we further broadened people’s understanding on the selection force acting on synonymous mutations. Synonymous mutations potentially change the tAI of a codon, and the changed tAI alters the translation rate of the codon. This chain reaction finally exposes synonymous mutations to natural selection.

Interestingly, we and others previously reported a kind of “context dependent” selection acting on synonymous mutations [18, 27]. Together with our current work (“context independent” selection pattern), it seems that the mRNA translation or splicing process is the main aspect that a synonymous mutation could impact. For other aspects like the change of DNA methylation status, the effect might not be direct and remains to be explored.

Although we studied the codon optimality and its selection patterns from the aspect of tAI and translation efficiency, our conclusion generally agreed with previous studies which claimed that G/C-ending codons are optimal and selected for. The observed GC3 is the relic of natural selection and it does not directly tell the functional relevance. We associated the pure genomic features with the potential function. Since 23 out of 32 high tAI codons are G/C-ending and 21 out of 29 codons are A/T-ending, our study has found a putative reason (higher translation rate) for the positive selection for optimal codons. Meanwhile, GC-biased gene conversion would mimic the pattern under natural selection for transnational efficiency, which means that gene conversion could lead to an overestimation of the optimality of C/G-to-A/T mutations [28]. Since the optimal codons in maize is biased toward C/G-ending ones, one cannot rule out the higher frequency of preferred codons is due to GC-biased gene conversion. For example, two derived mutation sites, T-to-C at site1 and G-to-A at site2, mean that in the population there are T and C alleles at site1, and G and A alleles at site2. If DAF_site1_ > DAF_site2_, then we would claim that T-to-C should be more favorable than G-to-A mutation according to our theory. However, this might also be caused by the biased gene conversion towards C allele at site1 and towards G allele at site2. We find a way to exclude this bias if we test our pattern among the A/T-ending codons alone or among the C/G-ending codons alone. We retrieved all A-to-T or T-to-A mutations, the tAI-up mutations have a median DAF of 0.388 and those tAI-down mutations have a median DAF of 0.301 (*p*-value = 2.9e-4, Wilcoxon rank sum test). Among all C-to-G or G-to-C mutations, the tAI-up mutations have a median DAF of 0.407 and those tAI-down mutations have a median DAF of 0.303 (p-value = 6.3e-8, Wilcoxon rank sum test). The A-T switches and C-G switches ensure that the comparison between the bi-allelic site is fair and not affect by the biased gene conversion. So that our conclusion is robust.

## Conclusions

Our observations reveal the selection force acting on synonymous mutations in the maize genome. They are not strictly neutral. The tAI-increasing mutations are positively selected while those tAI-decreasing ones undergo purifying selection. This selection strength might be relatively weak compared to the constrain on nonsynonymous mutations but should not be automatically ignored.

## Methods

### Data collection

CDS sequences were downloaded with the following links (also see Additional file 1: Figure S2), *Zea mays*: ftp://ftp.ensemblgenomes.org/pub/release-43/plants/fasta/zea_mays/cds/, *Daucus carota*: ftp://ftp.ensemblgenomes.org/pub/release-43/plants/fasta/daucus_carota/cds/, and *Triticum aestivum*: ftp://ftp.ensemblgenomes.org/pub/release-43/plants/fasta/triticum_aestivum/cds/.

The tRNA data were downloaded from GtRNAdb (Genomic tRNA database).

The RNA-seq data (of root samples) are downloaded with the following accession numbers: *Zea mays* (SRR8560815-SRR8560819), *Daucus carota* (SRR7641984-SRR7641993), *Triticum aestivum* (SRR8767835, SRR8767837).

### RNA-seq data analyses

We mapped the reads in the RNA-seq data to the reference sequences (coding sequences) using aligner Bowtie2 [29]. The longest isoform of each gene is chosen. The variant calling process is accomplished by software SAMtools [30]. The default parameters were used since they are suitable in most cases. The variant sites with allele frequency between 0.02 and 0.98 were defined polymorphic mutations. The reason for this is filtering step is to exclude some sequencing errors introduced to the mutation profile.

### Inferring the ancestral state of mutations

We use software “OrthoMCL” [31] to find orthologous genes. We have the CDS and protein sequences of *Zea mays* and other two outgroup species (carrot *Daucus carota* and wheat *Triticum aestivum*). We feed this software with the protein sequences of three species. Then OrthoMCL employs all-against-all blastp algorithm to identify orthologs between species and paralogs within species. It provides us the best-two-way hits, that is the reciprocal best similarity pairs. We only focus on the orthologous genes between species. For each ortholog group, we require it to contain three single gene of three species. For example, let M = maize gene, C = carrot gene and W = wheat gene, then group (M1, C1, W1) is wanted rather than group (M1, C1) or group (M1, M2, C1, W1).

Now that we have orthologous gene group (M1, C1, W1) resulted from OrthoMCL, we could use multiple alignment to align the CDS sequences of gene M1, C1 and W1. We use ClustalW [32] with parameters type = nucleotide and matrix = pam. With the CDS alignments, for each nucleotide in maize CDS, we could easily know the nucleotides at the orthologous site in wheat and carrot. Next, we should define the ancestral state of each position in maize CDS and decide whether a site should be included in the downstream variation analyses.

Our criteria for filtering the mutations were illustrated in Additional file 1: Figure S1. For both polymorphic or fixed mutations, the orthologous sites in wheat and carrot should have the same nucleotide. For example, in Additional file 1: Figure S1, for the two fixed and two polymorphic sites defined, the two outgroup species have the same nucleotide at the orthologous position. Particularly, for fixed mutations, the reference sequence in maize should be different from the sequence in wheat. For example, in Additional file 1: Figure S1, for the two fixed sites shown, the two outgroup species have the same nucleotide, but meanwhile different from the nucleotide in maize. We also excluded any polymorphic sites in the wheat and carrot CDS by mapping the RNA-seq data to their corresponding reference CDS. The *Zea mays* sites analyzed in our study have no polymorphisms in the orthologous sites in wheat and carrot.

### Calculation of tAI and CAI

Among the 61 possible anticodons (tri-nucleotides) generated from the “reverse complement” of the 61 sense codons, maize has 52 of them. Among the 20 amino acids (AAs), the number of possible anticodons carrying this AA ranges from 1 to 5. For example, Met, Trp, Tyr and Asn each has 1 corresponding anticodon while Arg, Leu and Ser each has 5 corresponding anticodons. Although there are only 52 types of anticodons in maize, there are as many as 1198 tRNA loci in the genome. On average, each specific anticodon has 23 tRNA copies and each AA has 2.6 corresponding anticodons in maize.

An *s*_*ij*_ value described the interaction between codon and anticodon [6]. The *s*_*ij*_ values defined in eukaryotes are suitable in our analyses [33]. For example, *s*_*G:U*_ = 0.7861, which has 15 cases of anticodon:codon pairs in maize; *s*_*I:C*_ = 0.4659, which has 9 anticodon:codon pairs; *s*_*I:A*_ = 0.9075, which has 9 anticodon:codon pairs; *s*_*U:G*_ = 0.6295, which has 13 anticodon:codon pairs. The remaining possible anticodon:codon pairs are Watson-Crick (or I:U) base-pairing which have *s*_*ij*_ = 0. From the cases of *s*_*G:U*_ and *s*_*U:G*_, we can tell that even for GU wobble base-pairing, the constraint would be different if G is put into the codon side or anticodon side.

Codon level tAI is irrespective of how many genes are used because it is determined by the copy numbers of corresponding tRNAs that could pair with a particular codon. In contrast, when calculating CAI [34], we require highly expressed genes. In our maize data, totally 19,493 highly expressed genes and 6,954,755 codons were used. In fact, CAI is a parameter used at the gene level (geometric mean of w_ij_ values described below). At codon level the parameters are RSCU (relative synonymous codon usage) or w_ij_, which is defined as the number of a codon divided by the maximum number among its synonymous codon(s). However, a mutation that increases the w_ij_ value of a codon would definitely increase the CAI of its host gene. Thus, define “a synonymous mutation that increases/decreases CAI” as the synonymous mutation that increases/decreases the frequency of the codon and we still use the term CAI to describe the codon level w_ij_ value.

### RNA secondary structure prediction

With a software from the Vienna RNA secondary structure packages [35] named RNALfold, we folded all the CDSs in maize (with default parameters). Given an input CDS sequence, the software would finally output the regions that could form secondary structures within this sequence. The regions with Z-score < 0 were regarded as the CDS regions with strong RNA structure. Next, for each CDS, we calculated the fraction of bases that are located in the structured regions (denoted as “structured%”). We use this “structured%” value to roughly represent the RNA folding efficiency or the tendency for an RNA to be folded into secondary structures.

### Statistical analysis and code availability

The statistical analyses were performed in the R environment (http://www.R-project.org/). Codes are available under request.

## Supplementary information

**Additional file 1:** **Figure S1.** Inference of ancestral state of the mutations. For polymorphic mutations (called from the deep sequenced RNA-seq data) and fixed mutations (obtained from the CDS alignment) in maize, the ancestral state is inferred from the orthologous nucleotide in wheat and carrot. Only the sites with identical bases between wheat and carrot are used. Take the case (of polymorphic mutations) shown in the graph as an example, the orthologous sites in two outgroups are C, and the reference genome of maize is also C, while in the RNA-seq data, both C and another nucleotide are detected. Furthermore, any potential polymorphic sites in wheat or carrot (that are detectable in RNA-seq in wheat or carrot) are discarded. **Figure S2.** Locations of the CDS sequence files and RNA-seq data of the plant species used in this study and the basic statistics of the maize genome. **Figure S3.** Fixed to polymorphic ratios of different mutation categories. Highly expressed and lowly expressed genes are classified. These mutation categories include nonsynonymous and nonsense mutations as well as the synonymous mutations that increase or decrease the tAI. **Figure S4.** DAF and MAF spectrum of polymorphic mutations of different mutation categories. Highly expressed and lowly expressed genes are classified. These mutation categories include nonsynonymous and nonsense mutations as well as the synonymous mutations that increase or decrease the tAI. *P* values are calculated from Wilcoxon rank sum tests. **Figure S5.** Relationships between tAI, GC content and RNA folding. a Spearman correlation between tAI and GC content of genes. *P* value <2e-16. b Spearman correlation between GC content and the percentage of RNA structured regions of genes. P value <1e-3. c GC contents in versus out of RNA structured regions. P value is calculated from Fisher’s exact test. d Spearman correlation between tAI and the percentage of RNA structured regions of genes. *P* value <1e-3. **Figure S6.** DAF and MAF spectrum of polymorphic mutations of different mutation categories. Genes with high or low GC contents are classified. These mutation categories include nonsynonymous and nonsense mutations as well as the synonymous mutations that increase or decrease the tAI. P values are calculated from Wilcoxon rank sum tests. **Figure S7.** Distance (bp) between the nearby “tAI-up” and “tAI-down” synonymous mutations within each gene. Each dot represents a gene. These genes are ranked by the median distance of the “tAI-up” and “tAI-down” mutations within them. Error bar represents 25 to 75% quantile. **Figure S8.** DAF and MAF spectrum of different mutation sets. For a “tAI-up” mutation, if its nearest mutation is also a “tAI-up” mutation, we name it “up near up”. If its nearest mutation is a “tAI-down” mutation, we name it “up near down”. Similarly, we define “down near up” and “down near down” mutations. No significant difference is detected using Wilcoxon rank sum test.

**Additional file 2: Table S1.** The list of single base synonymous mutations that increase the tAI.

## Data Availability

Reference sequences of CDS: *Zea mays*: ftp://ftp.ensemblgenomes.org/pub/release-43/plants/fasta/zea_mays/cds/Zea_mays.B73_RefGen_v4.cds.all.fa.gz *Daucus carota*: ftp://ftp.ensemblgenomes.org/pub/release-43/plants/fasta/daucus_carota/cds/Daucus_carota.ASM162521v1.cds.all.fa.gz *Triticum aestivum*: ftp://ftp.ensemblgenomes.org/pub/release-43/plants/fasta/triticum_aestivum/cds/Triticum_aestivum.IWGSC.cds.all.fa.gz The tRNA data (tRNA copy numbers in *Zea mays*) were downloaded from Genomic tRNA database (http://gtrnadb.ucsc.edu/GtRNAdb2/genomes/eukaryota/Zmays7/zeaMay7-tRNAs.fa). RNA-seq data: *Zea mays* (SRR7403478-SRR7403480 and SRR7403485- SRR7403487); *Daucus carota* (SRR7641984-SRR7641993); *Triticum aestivum* (SRR8767835, SRR8767837).

## Abbreviations

CDS: Coding sequence
tRNA: transfer RNA
mRNA: messenger RNA
CUB: Codon usage bias
CAI: Codon adaptation index
tAI: tRNA adaptation index
NGS: Next generation sequencing
