# Supplementary material for: Genome-wide analysis on the maize genome reveals weak selection on synonymous mutations
Source: BMC Genomics. 2020 Apr 29;21:333. doi: 10.1186/s12864-020-6745-3 (PMC7190201; doi:10.1186/s12864-020-6745-3)
Supplement: Supplementary file 1 — Additional file 1: Figure S1. Inference of ancestral state of the mutations. For polymorphic mutations (called from the deep sequenced RNA-seq data) and fixed mutations (obtained from the CDS alignment) in maize, the ancestral state is inferred from the orthologous nucleotide in wheat and carrot. Only the sites with identical bases between wheat and carrot are used. Take the case (of polymorphic mutations) shown in the graph as an example, the orthologous sites in two outgroups are C, and the reference genome of maize is also C, while in the RNA-seq data, both C and another nucleotide are detected. Furthermore, any potential polymorphic sites in wheat or carrot (that are detectable in RNA-seq in wheat or carrot) are discarded. Figure S2. Locations of the CDS sequence files and RNA-seq data of the plant species used in this study and the basic statistics of the maize genome. Figure S3. Fixed to polymorphic ratios of different mutation categories. Highly expressed and lowly expressed genes are classified. These mutation categories include nonsynonymous and nonsense mutations as well as the synonymous mutations that increase or decrease the tAI. Figure S4. DAF and MAF spectrum of polymorphic mutations of different mutation categories. Highly expressed and lowly expressed genes are classified. These mutation categories include nonsynonymous and nonsense mutations as well as the synonymous mutations that increase or decrease the tAI. P values are calculated from Wilcoxon rank sum tests. Figure S5. Relationships between tAI, GC content and RNA folding. a Spearman correlation between tAI and GC content of genes. P value <2e-16. b Spearman correlation between GC content and the percentage of RNA structured regions of genes. P value <1e-3. c GC contents in versus out of RNA structured regions. P value is calculated from Fisher’s exact test. d Spearman correlation between tAI and the percentage of RNA structured regions of genes. P value <1e-3. Figure S6. DAF and MAF spectrum [file 12864_2020_6745_MOESM1_ESM.pdf]

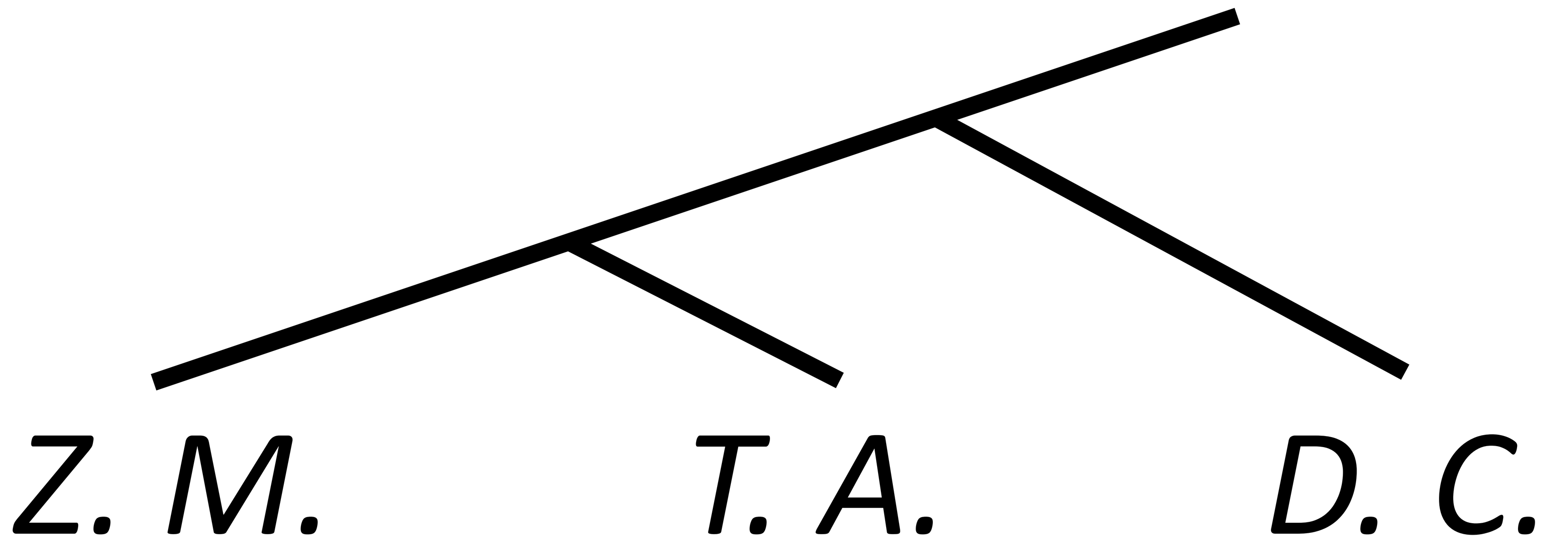

Z.M. : Zea mays.

T.A. : Triticum aestivum.

D.C. : Daucus carota.

ref.	**RNA-seq**			Mutation type in *Z.M.*
**C**	**C,A**	**C**	**C**	polymorphic
**C**	**C,T**	**C**	**C**	
**C**	**C**	**A**	**A**	fixed
**C**	**C**	**T**	**T**	
**C**	**C,A**	**A**	**C**	discarded
**C**	**C**	**T**	**C**	
**C**	**C,A**	**NA**	**C**	
**C**	**C**	**C**	**NA**	

NA = not available (indel/gap or unaligned)

Figure S1

Species	CDS sequence	RNA-seq data
Zea mays	ftp://ftp.ensemblgenomes.org/pub/release-43/plants/fasta/zea_mays/cds/	SRR7403478-SRR7403480, SRR7403485-SRR7403487
Triticum aestivum	ftp://ftp.ensemblgenomes.org/pub/release-43/plants/fasta/triticum_aestivum/cds/	SRR8767835, SRR8767837
Daucus carota	ftp://ftp.ensemblgenomes.org/pub/release-43/plants/fasta/daucus_carota/cds/	SRR7641984-SRR7641993

Species	Number of unique genes	Number of unique codons
Zea mays	39254	13958446

Number of mutations detected in the whole CDS regions

Type	Total	Synonymous	Non-synonymous	Nonsense
Fixed	843285	437056	400943	5286
Polymorphic	12041	4865	6875	301

Figure S2

Fixed/polymorphic ratio of mutations detected in the whole CDS regions

Type	Synonymous		Non-synonymous	Nonsense
	tAI up	tAI down		
Highly expressed genes	127.2	60.3	52.9	14.5
Lowly expressed genes	101.6	72.8	61.4	19.3

Figure S3

Frequency spectrum of the polymorphic mutations

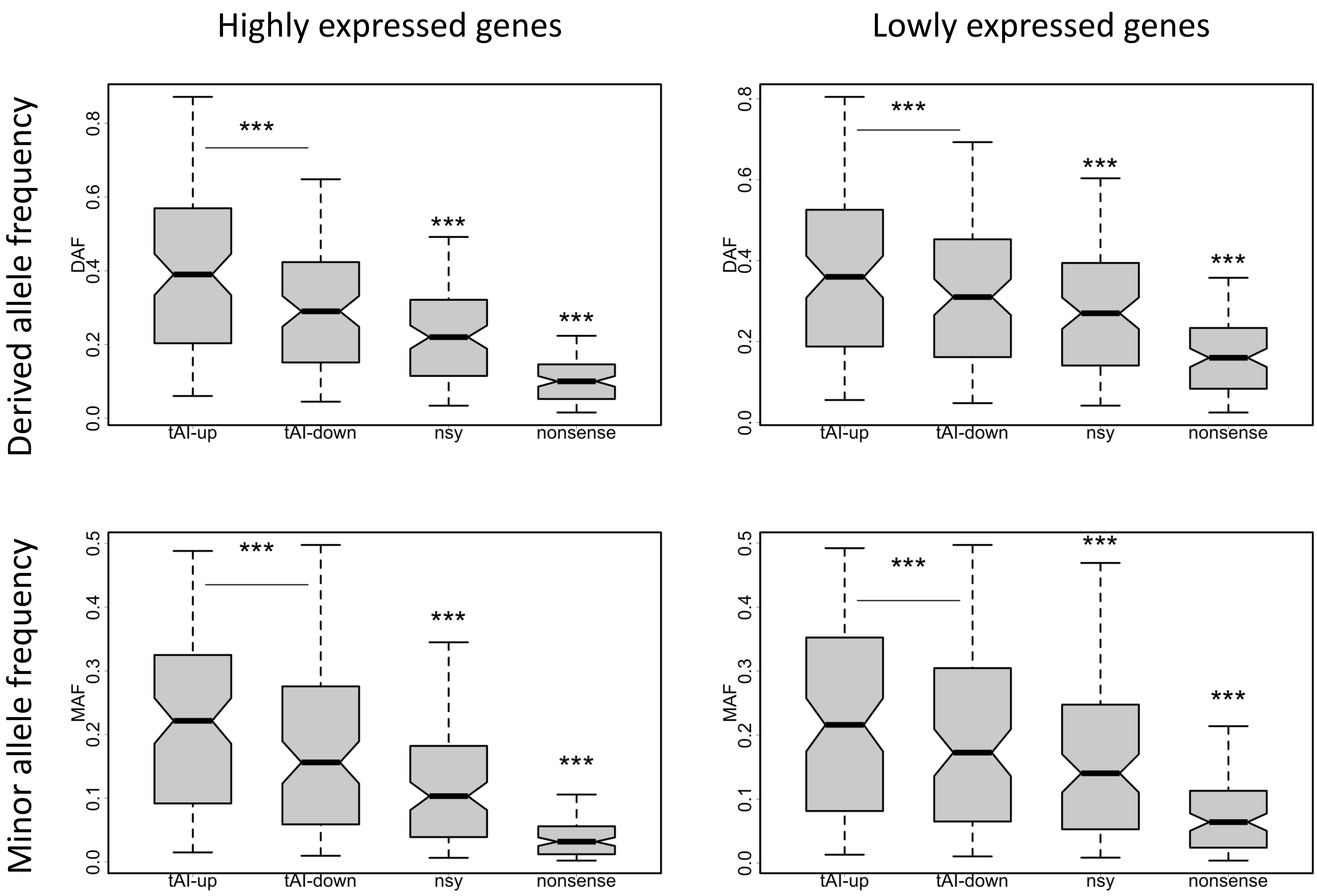

Figure S4

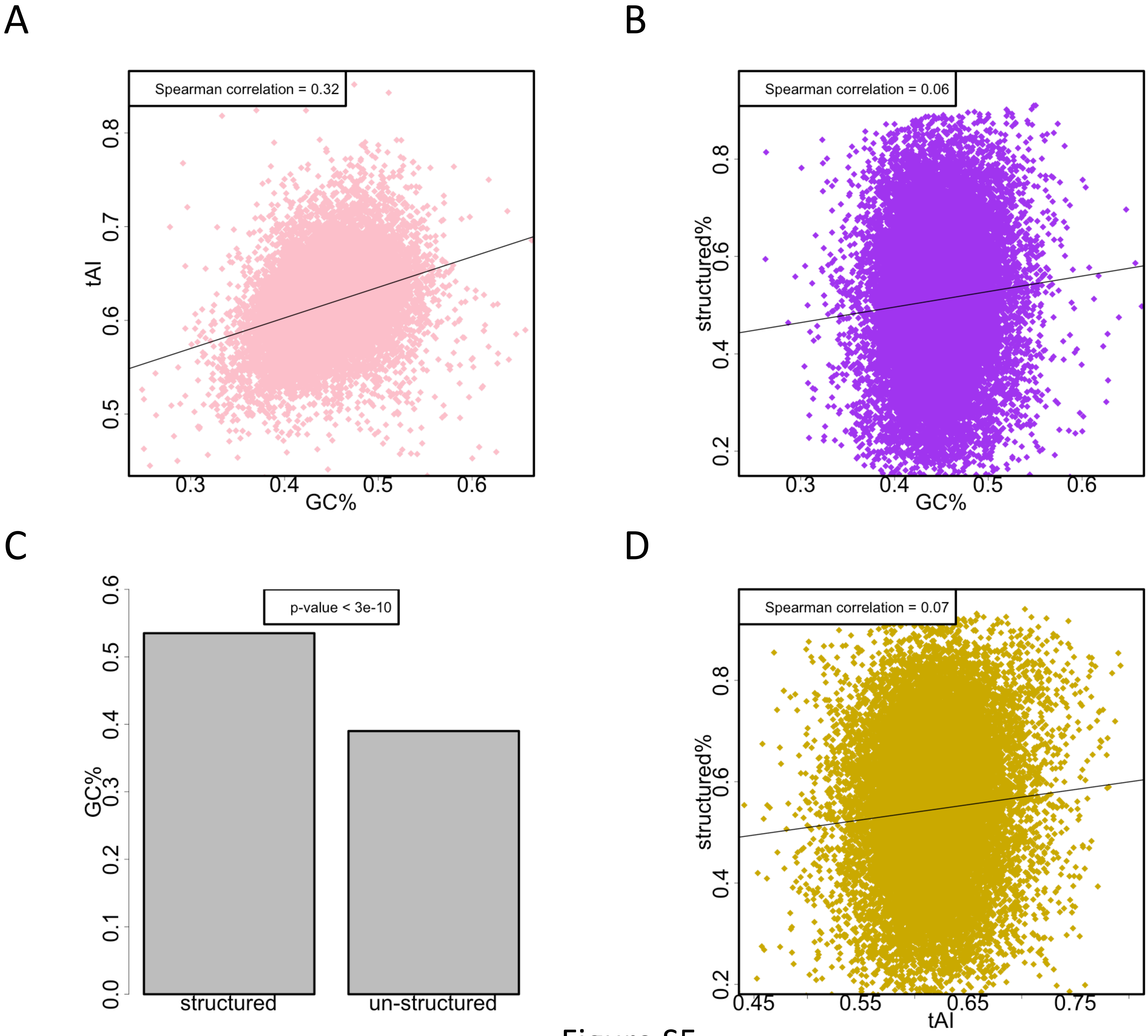
A
Spearman correlation = 0.32
tAI
GC%
B
Spearman correlation = 0.06
structured%
GC%
C
p-value < 3e-10
GC%
structured
un-structured
D
Spearman correlation = 0.07
structured%
tAI

Figure S5

Frequency spectrum of the polymorphic mutations

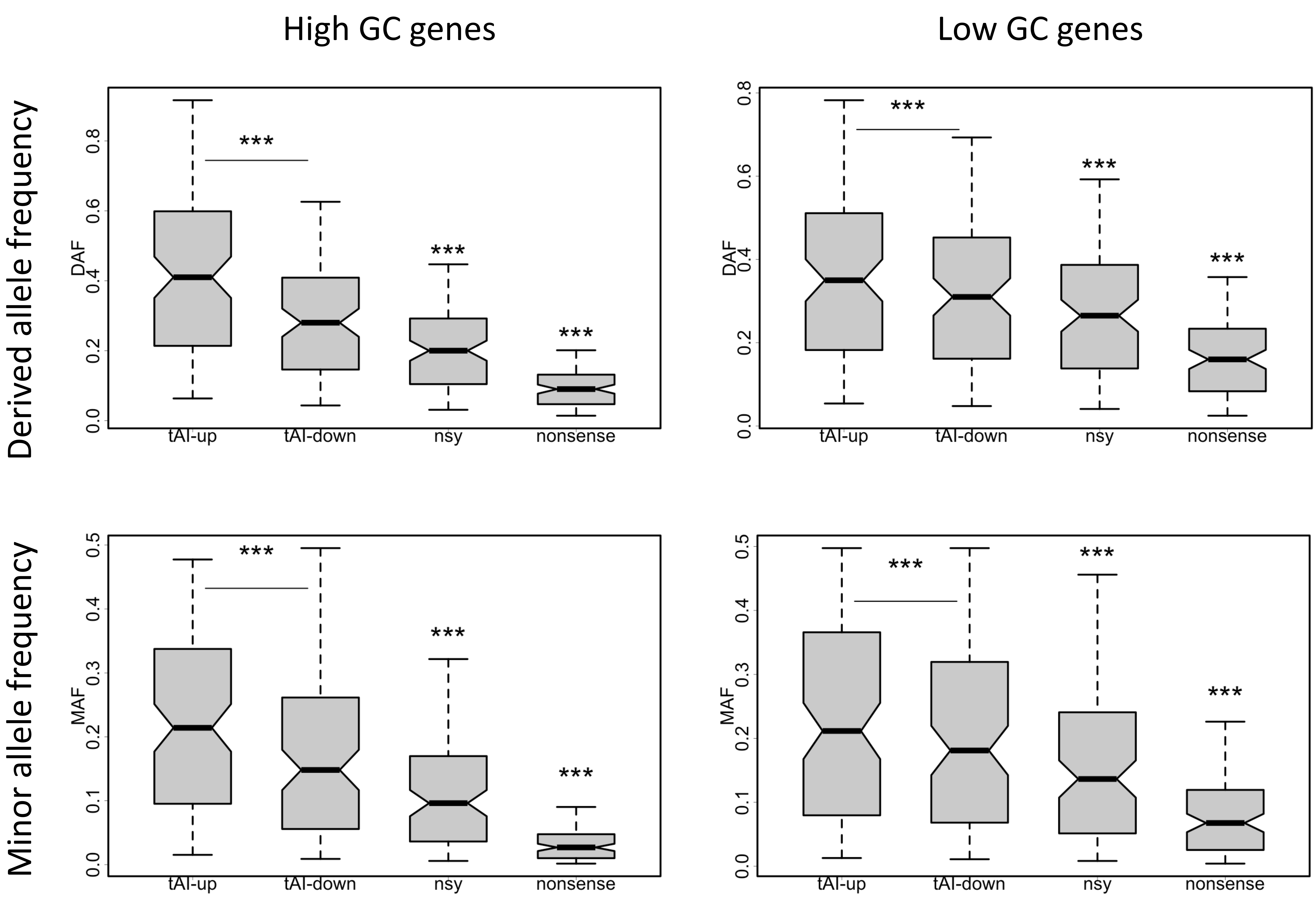

Figure S6

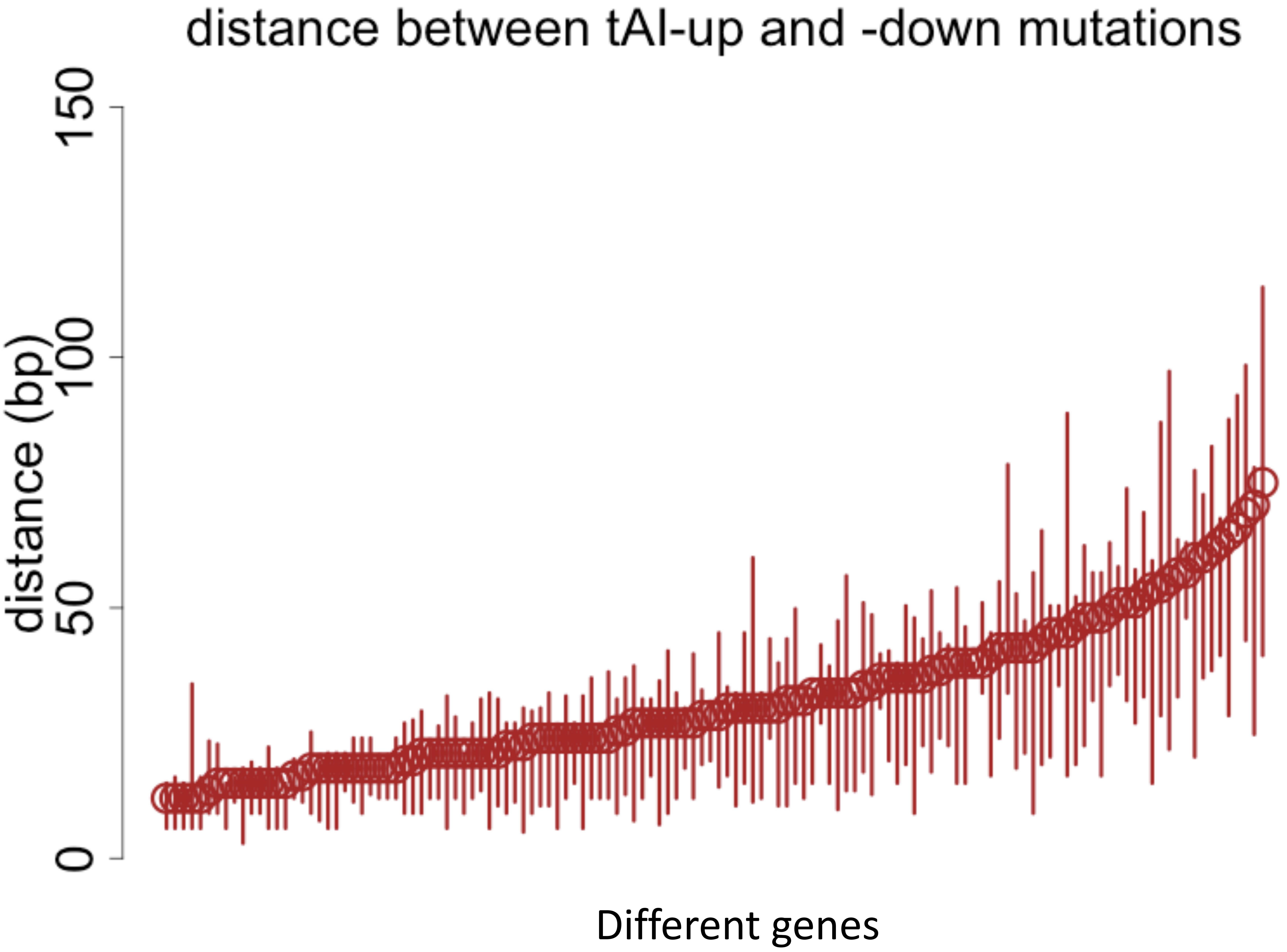
distance between tAI-up and -down mutations
distance (bp)
150
100
50
0
Different genes

Figure S7

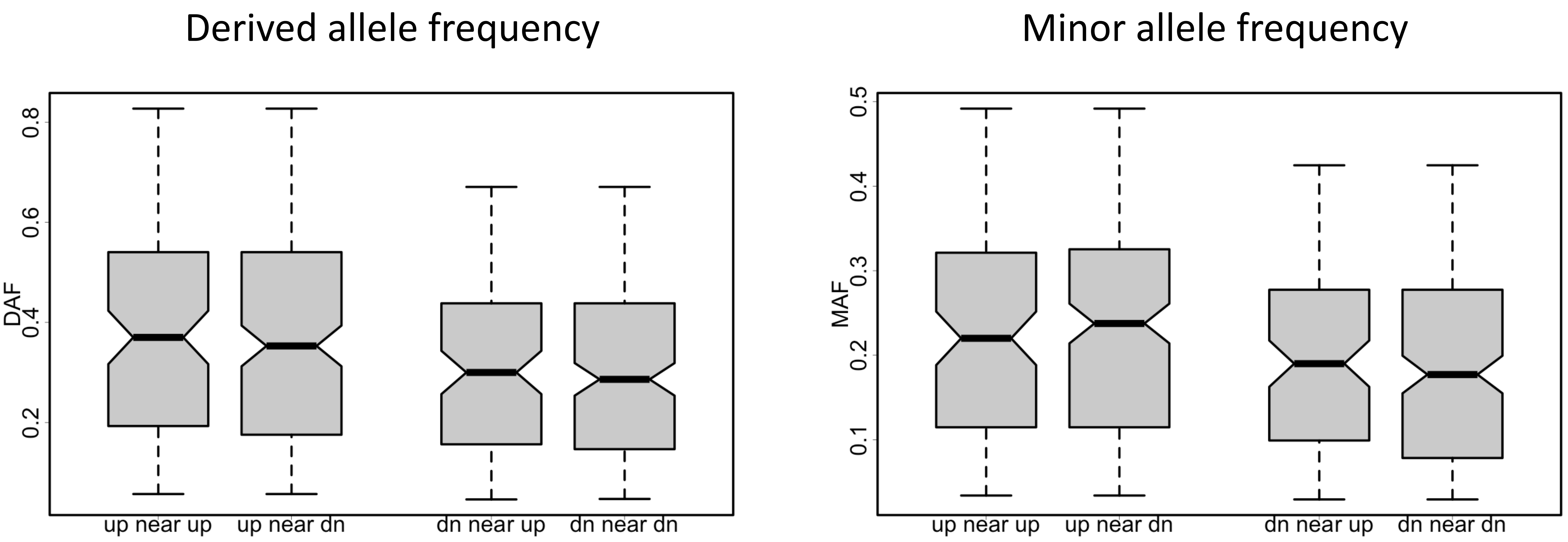
Derived allele frequency
Minor allele frequency
DAF
0.2
0.4
0.6
0.8
up near up
up near dn
dn near up
dn near dn
MAF
0.1
0.2
0.3
0.4
0.5
up near up
up near dn
dn near up
dn near dn

Figure S8
